# COMPARISON OF MUSCULAR ACTIVITY AND METABOLIC RESPONSE BETWEEN A NOVEL HANDLE-BASED AND A PUSH-RIM WHEELCHAIR IN A SIMULATED DAILY MOBILITY CIRCUIT

**DOI:** 10.2340/jrm.v58.44397

**Published:** 2026-01-15

**Authors:** Georgios ARONIS, Sebastian PFAU, Thomas ANGELI, Margit GFÖHLER

**Affiliations:** Research Unit of Biomechanics and Rehabilitation Engineering, Department of Engineering Design and Product Development TU Wien, Vienna, Austria

**Keywords:** wheelchair, electromyography, spirometry, -activities of daily living

## Abstract

**Objective:**

To compare the muscular activity and metabolic response between a novel handle-based wheelchair drive (KURT) and conventional push rim propulsion in a simulated daily mobility circuit.

**Design:**

Single-group comparative study between 3 wheelchair configurations.

**Participants:**

22 healthy individuals without prior wheelchair experience.

**Methods:**

Participants completed a multi-movement circuit including ramps, obstacle avoidance, and directional changes using KURT and 2 push-rim wheelchairs with different wheel sizes (small wheels, SW; large wheels, LW). Electromyographic data were collected bilaterally from 7 upper body muscles, and cardiopulmonary variables were continuously monitored.

**Results:**

Biceps brachii activity was significantly higher with KURT than with SW and LW for both arms (all *p* *<* 0.001), while triceps brachii and pectoralis major activity were significantly lower (all *p <* 0.001). Other monitored muscles showed smaller relative differences between configurations, often resulting in limited or no statistically significant effects. Metabolic demand was lower with KURT: heart rate, oxygen consumption, and carbon dioxide production were reduced compared with LW (all *p <* 0.05), while respiratory exchange ratio was unchanged and respiratory frequency was higher than with SW (*p <* 0.05).

**Conclusion:**

KURT appears to be a promising, more energy-efficient alternative to push-rim wheelchairs, reducing upper limb muscle demand and metabolic cost. These findings motivate studies in regular wheelchair users and longer-term use in daily living scenarios.

## LAY ABSTRACT

Push-rim wheelchairs are widely used but are associated with low energy efficiency and a high risk of upper limb overuse injuries. To address these issues, we developed a novel handle-based wheelchair aimed at reducing joint strain and improving propulsion efficiency. This study compared muscular and metabolic demands between handle-based and push-rim configurations during an everyday wheelchair course. 22 healthy, wheelchair-naive participants completed a course, including ramps, obstacle avoidance, and directional changes, using 3 wheelchair configurations: 1 handle-based and 2 push-rim setups with different wheel sizes. Electromyographic recordings and continuous cardiopulmonary data were collected. The handle-based system resulted in a redistribution of muscle activity, with reductions in several key propulsion muscles and an overall decrease in metabolic demand compared with push-rim propulsion. These findings suggest that the handle-based wheelchair may offer a more energy-efficient and joint-friendly alternative to conventional push-rim designs, with potential benefits for long-term mobility and upper limb health.

Upper extremity joint pain is the leading secondary complication associated with long-term manual wheelchair use, with over 70% of users experiencing shoulder pain (1). This is attributed to the repetitive nature of wheelchair propulsion, which can involve over 1,000 strokes per day (2, 3). This repetitive strain contributes to overuse injuries affecting the joints and surrounding musculature, sometimes resulting in chronic conditions (4). While electrically powered wheelchairs are often seen as a solution to musculoskeletal issues, they introduce their own complications. Specifically, prolonged reliance on powered mobility has been linked to reduced physical activity, diminished muscle mass, impaired circulation, and negative psychosocial effects, including lower self-esteem (5). This highlights the need for a better solution for individuals who are eligible for manual wheelchair use.

Hand bikes offer ergonomic benefits and improved mechanical efficiency (6); however, the addition of a front wheel significantly increases the device’s size, making it less suitable for indoor use. Inspired by this, our team developed a novel handle-based wheelchair (7) that offers a more ergonomic alternative to the push-rim wheelchair, which was used by 90% of users as of 2019 (8). This handle-based wheelchair (KURT) operates within a joint range closer to the physiological range of upper limb motion, thereby reducing excessive joint forces (9).

KURT’s effect on muscular activity and metabolic response was compared with that of the conventional push-rim wheelchair (8). The study used a test rig enabling 10 healthy participants to propel themselves at varying resistance levels. Peak muscular activity in the triceps brachii, posterior and anterior deltoid, and pectoralis major was significantly reduced, while activity in the biceps brachii increased with handle-based propulsion.

The same test rig was used to assess the impact of the handle-based mechanism on metabolic variables such as heart rate (HR), oxygen uptake (V_o2_), ventilation volume (*V*_e_), respiratory exchange ratio (RER), and gross mechanical efficiency (GME) (10). For healthy participants, all variables showed a statistically significant reduction when using handle-based propulsion, except HR, which increased slightly at lower resistance levels.

After completing the experiments, user feedback from wheelchair participants was gathered, leading to the development of an improved prototype to enhance usability (8, 10). Two major modifications were made: the addition of a reclining propulsion module to facilitate easier transfers, and the replacement of the hydraulic brakes with a mechanism allowing forward, reverse, and braking through handlebar rotation.

Following these changes, the new, improved wheelchair was tested in a circuit simulating daily wheelchair movement patterns alongside 2 configurations of the conventional push-rim model. The aim of this study was to evaluate differences in muscular activity and metabolic response between the novel handle-based wheelchair and the conventional push-rim wheelchair during a simulated daily mobility circuit. It was hypothesized that users would show reduced metabolic cost and overall reduced or comparable muscular activity, except for the biceps, when using the handle-based wheelchair, with a different distribution of muscle activity that is consistent with prior studies (8, 10).

## METHODS

### Propulsion devices

**Fig. 1:**
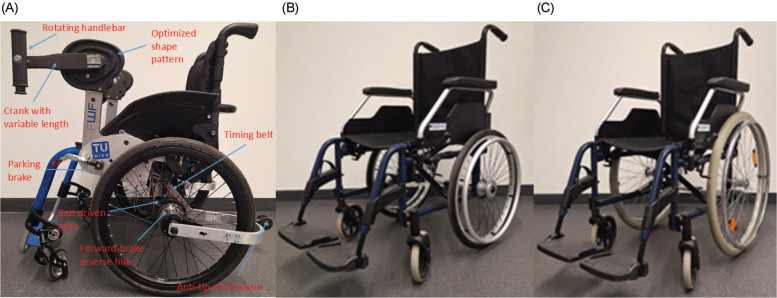
(A) KURT novel wheelchair, (B) push-rim 20″ wheels, and (C) push-rim 24″ wheels.

The new KURT wheelchair was designed and fabricated at the Institute of Engineering Design and Product Development, TU Wien. The propulsion system was mounted on an Ottobock Ventus wheelchair frame (Ottobock, Duderstadt, Germany). As shown in Fig. 1, by rotating the handlebar the arms of the user are forced to follow the optimized shape pattern hence constraining the upper limbs of the user to a range of motion that induces less stress on the joints. Housed within the component defined by the shape pattern is a driving gear, which transfers rotational motion to the wheels through a timing belt mechanism. A mechanism integrated within the wheel hub enables forward propulsion when the handlebar is rotated forwards, backward motion when rotated in the opposite direction, and braking when the handlebars are locked in place after briefly reversing the crank to engage the brake. A parking brake system was implemented to lock the wheelchair in position when required, while an anti-tip mechanism was integrated to prevent overturning and enhance user safety. For comparison with the KURT system, a conventional push-rim wheelchair was selected for evaluation: the Meyra Budget 9050 model (Meyra, Porta Westfalica, Germany). Two wheelchair configurations were tested: 1 equipped with the original 24″ wheels and another with customised 20″ wheels. The inclusion of the 20″ configuration was necessary because the Ottobock Ventus frame used to mount the KURT propulsion system was originally designed for 20″ wheels. Testing both wheel sizes allowed for an objective comparison of propulsion performance by removing the mechanical advantage of the larger 24″ wheels.

### Participants

A total of 22 healthy men participated in the study. Participant characteristics are reported as mean (standard deviation [SD]): age 27.8 (5.4) years, height 180.0 (5.1) cm, and body mass 78.0 (9.1) kg. One participant was left-handed. None of the participants reported current upper limb conditions that could affect propulsion (e.g., ongoing pain, unresolved injury, recent surgery, or diagnosed musculoskeletal or neurological disorders of the shoulder or arm). None had prior experience using a manual wheelchair. The sample size was guided by previous studies investigating wheelchair propulsion under comparable conditions (6, 8). Drawing on these examples and employing a within-subject design, a sample of 22 healthy participants was considered appropriate to investigate performance differences across wheelchair configurations. Only men were included to minimize sex-related variability in anthropometrics, muscle mass distribution, and cardiopulmonary capacity in this initial prototype evaluation. Before the initiation of the experiment, participants received and signed an informed consent form outlining all experimental procedures and the use of their data. The form was reviewed and refined with input from TU Wien’s Ethics Committee.

### Task

**Fig. 2:**
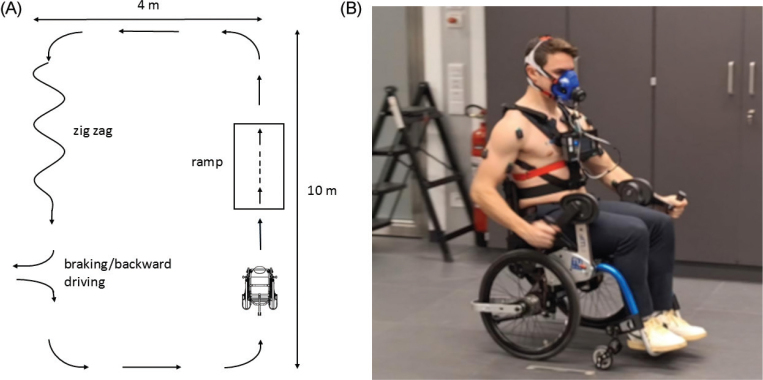
(A) Plan of circuit followed by participants, (B) participant executing the task with KURT.

All participants were tested individually in the same laboratory environment using an identical protocol to ensure consistency across the 3 wheelchair configurations. The experimental task required participants to complete a circuit established within the motion analysis laboratory of our institute. The circuit illustrated in Fig. 2A incorporated various types of movement, including ascending and descending a 5° ramp, zig-zagging to avoid obstacles, braking, reversing, and turning. Participants were instructed to complete 3 clockwise and 3 anticlockwise rotations to counterbalance the effect of the increased arm strokes generated by the limb positioned on the outer side of the curve. Participants were instructed to complete the circuit at a self-selected pace that they found comfortable. The procedure was executed 3 times, once with KURT (Fig. 2B), once with the large wheels (LW) push-rim configuration, and once with the small wheels (SW) push-rim configuration. The order of testing configurations was partially counterbalanced: participants started either with LW or SW, then completed the circuit with KURT, and finally performed the remaining push-rim configuration. This sequence accommodated the wheel changes required between LW and SW while limiting systematic order effects. Between tests, participants were permitted to rest for several minutes until their metabolic variables returned to baseline values, which were measured before the start of the first trial.

Before the experiment, the participants were asked to prepare by performing a brief standardized warm-up routine consisting of 3–5 min of dynamic upper-body exercises (neck, shoulder, elbow, and wrist movements) to decrease the risk of injury. After that, they were asked to familiarize themselves with all 3 wheelchair configurations by driving them for several minutes along the route. In addition to this, participants were requested to be rested and avoid drinking alcohol and strenuous exercise for at least 24 h before the experiment.

### Data collection

Electromyographic (EMG) data were recorded using wireless Delsys Trigno Avanti and Mini surface EMG sensors (Delsys Inc, Natick, MA, USA) at a sampling rate of 1260Hz. EMGworks acquisition software (Delsys Inc, Natick, MA, USA) was used to collect the data. EMG activity of the biceps brachii, triceps brachii, anterior deltoid, latissimus dorsi, flexor carpi radialis, pectoralis major, and trapezius lower fibres was recorded for both the right and left side of the body. Trigno Mini sensors were used for the pectoralis major and the latissimus dorsi, as their smaller size minimized interference with the spirometer mounting harness and the wheelchair. Before sensor placement, the skin at each site was cleaned with 70% isopropanol to improve sensor–skin contact. Sensor placement for the biceps brachii, triceps brachii, anterior deltoid, and trapezius lower fibres followed the SENIAM guidelines (11), while sensor locations were chosen according to published recommendations for the latissimus dorsi (12), pectoralis major (13), and flexor carpi radialis (14). Maximum voluntary contraction (MVC) was recorded before the test by performing 3 isometric contractions. Participants were instructed to contract the target muscle as forcefully as possible for 5 s, with verbal encouragement, followed by a 30 s rest. This process was repeated twice more, and the peak activity recorded during this period was considered the MVC. Noisy or unreliable EMG recordings from specific muscles were excluded from the analysis. Missing data were handled by analysing only complete and reliable datasets for each participant.

Cardiopulmonary data were obtained with a portable Cosmed-K5 ergo spirometer (Cosmed, Rome, Italy). Metabolic variables recorded included oxygen consumption (*V_O_*_2_, ml·min^-1^·kg^-1^), carbon dioxide production (*V_CO_*_2_, ml·min^-1^·kg^-1^), respiratory exchange ratio (RER, –) and respiratory frequency (Rf, breaths·min^-1^). These variables were recorded every 10 s in mixing-chamber mode. The spirometer was calibrated prior to each participant trial. Heart rate (HR, bpm) was measured using a Bluetooth Smart and ANT+ dual chest strap monitor (G. Pulse International Co, Taichung City, Taiwan).

### Data processing and analysis

The raw EMG signals regarding both MVC and the task activity were initially offset-corrected to remove baseline drift. Consequently, a bandpass filter of 20 to 450 Hz frequency range was implemented to remove signals not related to muscular activity. A moving root mean square (RMS) envelope, which quantifies the amplitude of the EMG signal over time, was then computed using a 250 ms window with 50% overlap. Finally, it was normalized by dividing it by the MVC to get the mean RMS activity for all muscles during the task for the different wheelchair configurations. Test results are presented as mean (SD). Python (https://www.python.org/) was used to analyse the signals and assess the statistical significance between the 3 wheelchair configurations. The Wilcoxon signed-rank test was used to inspect whether there is a statistically significant difference between the muscular activity and metabolic variables mean among the 3 configurations because the data were not normally distributed. The within-subject design controlled for inter-individual variability (e.g., strength, coordination, anthropometrics), as each participant completed the task with all configurations. No subgroup, interaction, or sensitivity analyses were conducted. A *p*-value *<* 0.05 was considered statistically significant (*), *p <* 0.01 as very significant (**), and *p <* 0.001 as highly significant (***). All relative differences are calculated in respect of KURT configuration.

## RESULTS

**Fig. 3:**
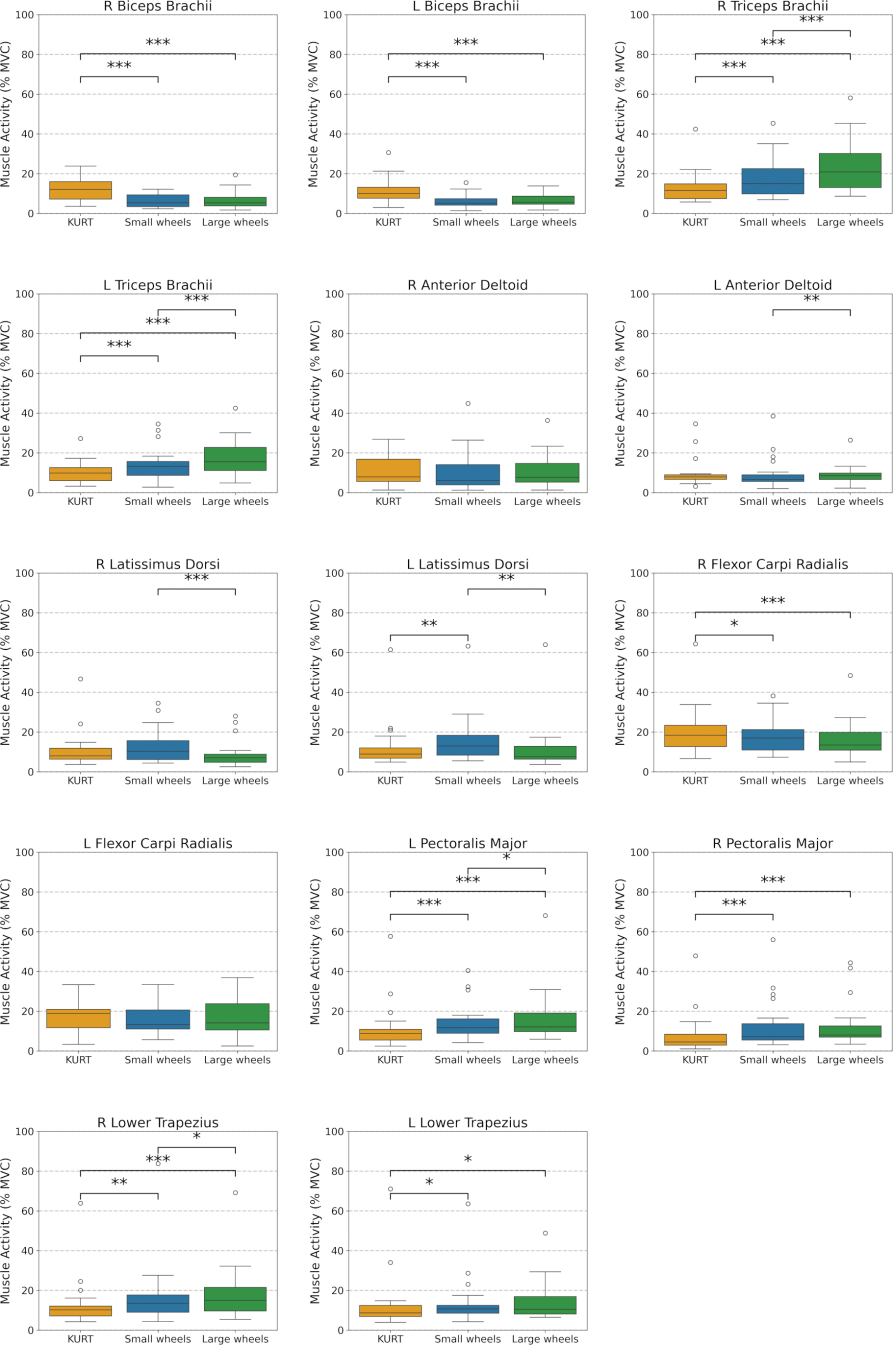
Comparison of mean, normalized muscle activity (%MVC) across KURT, Small Wheels, and Large Wheels configurations. Box plots show the median, IQR, and minimum and maximum values across all subjects.

**Fig. 4:**
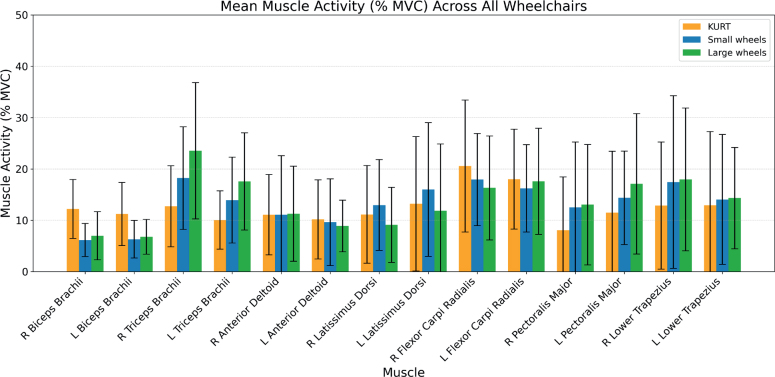
Mean and standard deviation of normalized muscle activity (%MVC), averaged across all subjects, for the KURT, Small Wheels, and Large Wheels configurations.

**Fig. 5:**
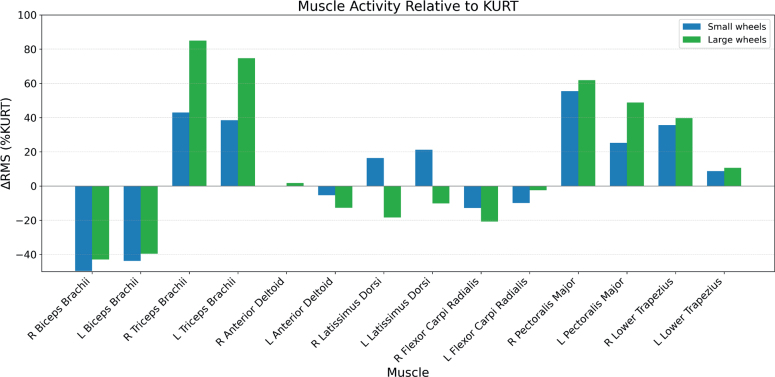
Relative difference in mean normalized muscle activity (%MVC) between configurations, across all subjects.

**Fig. 6:**
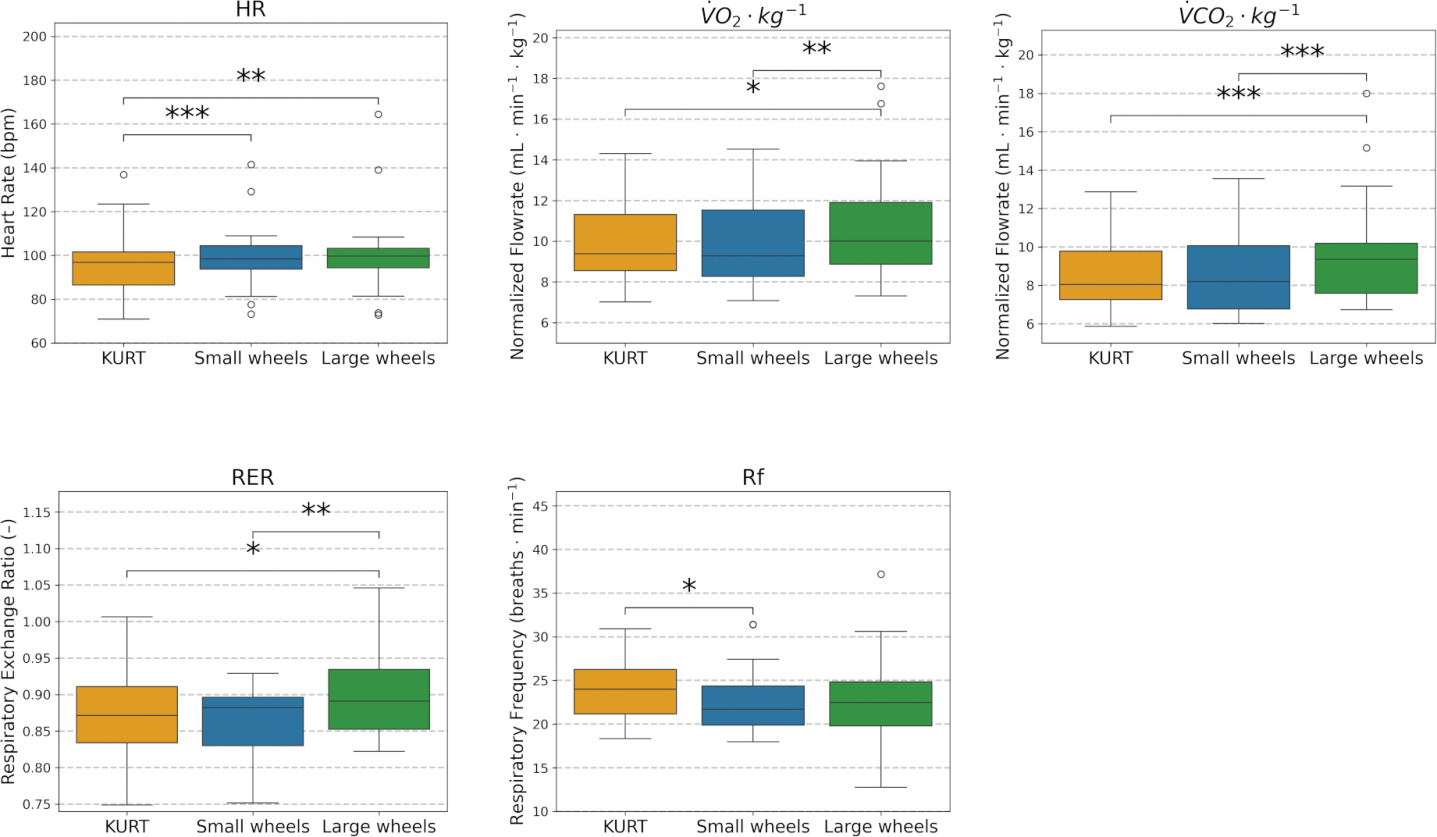
Comparison of metabolic response across KURT, Small Wheels, and Large Wheels configurations. Box plots show the median, IQR, and minimum and maximum values across all subjects.

All 22 participants completed all testing configurations and were included in the final analysis. Muscle activity (%MVC) and metabolic variables differed between propulsion modes, with the largest EMG changes in biceps brachii, triceps brachii, and pectoralis major, and generally lower physiological demand when using KURT compared with both push-rim wheelchairs (Figs 3–6, Tables I–II).

**Table I:** Mean (standard deviation) for all muscles for KURT (K), Large Wheels (LW), and Small Wheels (SW) configurations. Number of subjects with valid EMG recording (*n*), *p*-value calculated with Wilcoxon signed-rank test

Muscle	Mean (SD)(K) (% MVC)	Mean (SD)(LW) (% MVC)	Mean (SD)(SW) (% MVC)	ΔRMS % KK vs LW (*p*-value)	ΔRMS % KK vs SW (*p*-value)	*n* (K/SW/LW)
R Biceps Brachii	12.2 (5.7)	7.0 (4.7)	6.1 (3.2)	–42.6 (*<* 0.001)	–50.0 (*<* 0.001)	22/21/21
L Biceps Brachii	11.2 (6.1)	6.8 (3.4)	6.3 (3.7)	–39.3 (*<* 0.001)	–43.8 (*<* 0.001)	21/20/21
R Triceps Brachii	12.7 (7.9)	23.5(13.3)	18.2 (10.0)	85.0 (*<* 0.001)	43.3 (*<* 0.001)	22/22/22
L Triceps Brachii	10.1 (5.7)	17.6 (9.5)	13.9 (8.4)	74.3 (*<* 0.001)	37.6 (*<* 0.001)	22/21/21
R Anterior Deltoid	11.1 (7.8)	11.3 (9.3)	11.1 (11.5)	1.8 (0.720)	0.0 (0.821)	18/15/17
L Anterior Deltoid	10.2 (7.7)	8.9 (5.0)	9.6 (8.4)	–12.7 (0.711)	–5.9 (0.212)	19/19/20
R Latissimus Dorsi	11.1 (9.5)	9.1 (7.3)	13.0 (8.8)	–18.0 (0.325)	17.1 (0.090)	20/20/18
L Latissimus Dorsi	13.2 (13.1)	11.9 (13.0)	16.0 (13.0)	–9.8 (0.141)	21.2 (*<* 0.01)	17/19/19
R Flexor Carpi Radialis	20.6 (12.9)	16.3 (10.1)	17.9 (9.0)	–20.9 (*<* 0.001)	–13.1 (*<* 0.05)	19/20/16
L Flexor Carpi Radialis	18.0 (9.7)	17.6 (10.3)	16.2 (8.5)	–2.2 (0.203)	–10.0 (0.426)	9/11/10
R Pectoralis Major	8.0 (10.4)	13.0 (11.7)	12.5 (12.7)	62.5 (*<* 0.001)	56.3 (*<* 0.001)	21/21/21
L Pectoralis Major	11.5 (12.0)	17.1 (13.7)	14.4 (9.1)	48.7 (*<* 0.001)	25.2 (*<* 0.001)	22/21/22
R Lower Trapezius	12.9 (12.4)	18.0 (13.9)	17.4 (16.8)	39.5 (*<* 0.001)	34.9 (*<* 0.01)	22/21/20
L Lower Trapezius	12.9 (14.4)	14.3 (9.9)	14.0 (12.7)	10.9 (*<* 0.05)	8.5 (*<* 0.05)	22/22/21

**Table II:** Mean (standard deviation) for metabolic variables for KURT (K), Large Wheels (LW), and Small Wheels (SW) configurations

Variable	Mean (SD)(K)	Mean (SD)(LW)	Mean (SD)(SW)	Δ% KK vs LW (*p*-value)	Δ% KK vs SW (*p*-value)
HR (1/min)	95.94 (15.28)	100.75 (19.40)	98.48 (15.43)	5.0% (*<* 0.01)	2.6% (*<* 0.001)
V*_o_*_2_(ml·min^-1^·kg^-1^)	9.94 (2.15)	10.75 (2.69)	10.06 (2.23)	8.2% (*<* 0.05)	1.2% (0.726)
V*_Co_*_2_ (ml·min^-1^·kg^-1^)	8.64 (2.13)	9.68 (2.76)	8.71 (2.17)	12.0% (*<* 0.001)	0.8% (0.610)
RER (–)	0.87(0.06)	0.90 (0.05)	0.86 (0.05)	3.4% (*<* 0.05)	–1.1% (0.156)
Rf (breaths· min^-1^)	23.73 (3.26)	22.70 (5.32)	22.65 (3.73)	–4.3% (0.129)	–4.6% (*<* 0.05)

Rf (breaths·min^-1^) is the respiratory frequency, HR (bpm) is the heart rate, RER (–) is the respiratory exchange ratio, *V_o_*_2_ (ml·min^-1^·kg^-1^) is the volume of oxygen inspired and *V_Co_*_2_ (ml·min^-1^·kg^-1^) is the volume of carbon dioxide expired during the task (subjects with valid results *n* = 22), *p*-value calculated with Wilcoxon signed-rank test.

### Muscular activity

Inter-subject variability and mean normalized muscle activity (%MVC) across the 3 configurations are shown in Fig. 3 (box plots of median, interquartile range (IQR), minimum and maximum) and Fig. 4 (group means with standard deviations). Relative differences in mean activity between KURT and the 2 push-rim configurations are illustrated in Fig. 5, and the corresponding numerical values (mean, SD, ΔRMS, *p*-values and *n*) are reported in Table I.

There was a significant effect of propulsion mode on EMG activity for several upper-limb muscles (see Table I). Biceps brachii, triceps brachii, and pectoralis major showed large, statistically significant differences between configurations (see Figs 4 and 5, Table I). Biceps activity on both sides was substantially lower with the push‑rim wheelchairs than with KURT, with reductions of ~40–50% for SW and ~40% for LW relative to KURT (all *p <* 0.001; see Table I). Triceps activity showed the opposite pattern, with increases of ~40% for SW and ~75–85% for LW compared with KURT on both sides (all *p <* 0.001; Table I). Pectoralis major activity also increased with push‑rim propulsion, by 25.2% (left) and 56.3% (right) with SW and by 48.7% (left) and 62.5% (right) with LW relative to KURT (all *p <* 0.001; Table I).

For the remaining muscles, propulsion‑mode effects were smaller and differed between individual muscles (see Table I). Anterior deltoid activity showed only small, non‑significant differences between configurations on both sides. Latissimus dorsi activity was highest with SW, followed by KURT and lowest with LW; this ordering reached statistical significance for the left latissimus dorsi between KURT and SW (+21.2%, *p <* 0.01; Table I). Flexor carpi radialis activity tended to be lower with the push‑rim wheelchairs than with KURT, with a significant reduction on the right side (K vs LW: −20.9%, *p <* 0.001; K vs SW: −13.1%, *p <* 0.05; Table I). Lower trapezius activity was higher with push‑rim propulsion, with significant increases on the right side (K vs LW: +39.5%, *p <* 0.001; K vs SW: +34.9%, *p <* 0.01), whereas left‑side differences, although statistically significant, were small in magnitude (Table I).

### Metabolic response

Metabolic responses for the 3 configurations are reported numerically in Table II and illustrated as inter‑subject distributions in Fig. 6. Most metabolic variables were lower when using the KURT configuration than when using the push‑rim wheelchairs (Fig. 6, Table II). RER showed only a small, non‑significant difference between SW and KURT (Δ% −1.1%, *p* = 0.156), whereas respiratory frequency was reduced with SW (Δ% −4.6%, *p <* 0.05) and with LW (Δ% −4.3%, *p* = 0.129) relative to KURT (Table II). Heart rate was higher during push‑rim propulsion, corresponding to an increase of 2.6% with SW (*p <* 0.001) and 5.0% with LW (*p <* 0.01) compared with KURT (Table II).

## DISCUSSION

The aim of this study was to compare muscular activity and metabolic response during propulsion between a conventional push-rim wheelchair and a novel optimized ergonomic wheelchair (KURT) that can be used as a substitute, especially for people with chronic upper limb joint pain. The comparison task involved navigating a laboratory circuit designed to simulate everyday wheelchair manoeuvres and challenges encountered by wheelchair users in daily life. It was hypothesized that users would show reduced metabolic cost and overall reduced or comparable muscular activity, except for the biceps, when using the handle-based wheelchair, with a different distribution of muscle activity consistent with earlier work (8, 10). This hypothesis was largely supported by the results of this study: compared with KURT, push-rim propulsion was associated with lower biceps activity but higher triceps, pectoralis major, and lower trapezius activity, whereas KURT showed similar anterior deltoid, latissimus dorsi, and flexor carpi radialis activity and a lower overall cardiopulmonary load than both push-rim configurations.

Biceps activity was reduced by ~40–50% when push-rim wheelchairs were used. This aligns with findings from a study that employed a stationary test rig and compared KURT and push-rim wheelchair propulsion under 2 resistance levels, reporting a ~70% decrease in peak biceps activity with push-rim (8). The electromyographic activity of upper limb muscles was also investigated in a study comparing push-rim and crank-propelled wheelchairs, such as KURT, which found a statistically significant ~30% reduction in mean biceps activity with push-rim propulsion among able-bodied adults (15).

Triceps activity was ~40% higher with SW and ~75–85% higher with LW compared with KURT. Similarly, a relative decrease in peak activity of ~50–60% was observed with KURT in previous work (8). This difference primarily arises because push-rim propulsion requires users to apply force tangentially to the handrim through elbow extension, primarily driven by the triceps. During the recovery phase, elbow flexion occurs (activating the biceps) without producing force on the rim, reducing biceps load (16). In contrast, crank propulsion, such as in KURT, engages both triceps and biceps more continuously, distributing load more effectively across the cycle. Previous research indicated that in KURT, biceps are active for ~50–65% of the cycle and triceps for ~30-55%, depending on resistance, while in push-rim propulsion, biceps are active for only ~15–25% and triceps for ~70%, creating a higher muscular imbalance (8).

The pectoralis major also showed high statistically significant differences between KURT and the push-rim configurations. With SW, muscular activity increased by 25.2% (left side) and 56.3% (right side) relative to KURT. For LW, the differences were even greater: 48.7% (left side) and 62.5% (right side). A previous study reported an increase in peak pectoralis major activity of over 150% with push-rim propulsion (10). This may be attributed to the internal shoulder rotation required to align the palm with the handrim, which is greater compared with KURT (9). This explains the large difference between the cases as pectoralis major is a main contributor to shoulder internal rotation (17). The anterior deltoid and clavicular portion of the pectoralis major are the primary shoulder flexors at low flexion/extension angles in the sagittal plane (18). According to a previous study (9), during push-rim wheelchair propulsion, the arm extends up to 60° and flexes to around 20°. This is similar to values reported in a former investigation that tested 6 participants and identified joint angles ranging from 70° extension to 20° flexion during push-rim propulsion (19). In KURT, the range of extension is comparable, but flexion remains below 10°, as shown by simulation results (9).

Depending on resistance, the anterior deltoid is activated for ~50–60% of the propulsion cycle in KURT (8). The combination of this factor and the comparable range of motion likely explains the small average deviations observed between the activity of the anterior deltoid in KURT and the 2 push-rim configurations. However, a substantial difference exists between the mean anterior deltoid activity observed in this study (8.7–11.3% MVC) and the peak values previously reported (28.7–80.2% MVC) across various resistances (8). This may be due to the lower resistance in our protocol. Rolling resistance power depends on both wheel and flooring type (20). In our setup, the estimated rolling resistance power for the LW configuration, which represented the most demanding condition, was approximately 16 W, which is consistent with earlier research involving slightly lighter participants moving at similar speeds (21). The literature also indicates that lower resistance yields smaller flexion/extension angles and lower resultant push-rim forces (22). These factors likely explain the lower %MVC values for anterior deltoid observed here compared with prior studies (8).

The right lower trapezius displayed significantly higher activity with push-rim propulsion, while the left side increase was not statistically significant. Although no specific studies have evaluated the lower trapezius in push-rim propulsion, its function in scapular upward rotation (23), which stabilizes the scapula during the push phase (24), may account for this difference. Stabilization of the scapula supports shoulder flexion and abduction, which are crucial for effective rim propulsion. Crank propulsion, as in KURT, involves limited shoulder abduction, reducing scapular upward rotation and therefore diminishing the demand for elevated lower trapezius activity during propulsion.

Latissimus dorsi activity was greater in SW than in LW. Propelling with a smaller rim generally requires more forward trunk lean, increasing the need for latissimus dorsi recruitment (25). This muscle helps stabilize the trunk and spine, assists shoulder extension and adduction, and maintains scapular control. In KURT, the latissimus dorsi contributes during the backward and downward movement of the crank handle and supports shoulder and trunk stabilization throughout the propulsion cycle. A statistically significant difference was found for the left latissimus dorsi between KURT and SW, with lower activity in KURT. However, the right-side difference was not significant and justifies further investigation in future work.

Flexor carpi radialis showed increased activity in KURT compared with SW and LW on both sides, though significance was reached only on the right, likely due to a higher number of valid EMG datasets. This may be because users maintain continuous hand contact with the crank handles in KURT, requiring constant wrist stabilization and grip support. In contrast, push-rim propulsion involves intermittent contact with the rim, reducing the duration and intensity of muscle activity. However, this finding was unexpected, as previous work showed more pronounced wrist flexion and radial deviation, controlled by this muscle during push-rim propulsion (9).

HR significantly decreased with KURT compared with LW (*p <* 0.01) and SW (*p <* 0.001), with reductions of 5.0% and 2.6%, respectively. A previous study reported a 1% increase in HR at 15 W during submaximal testing among 9 able-bodied individuals (10). At 35 W, however, HR was 3% lower with KURT. *V_O_*_2_, *V_CO_*_2_ and RER followed similar trends in our results, although differences in *V_O_*_2_, and *V_CO_*_2_ between KURT and SW were not statistically significant. That earlier study reported a 10% *V_O_*_2_ reduction with KURT, closely matching our observed 8.2% decrease compared with LW (10). It also found a 4.8% decrease in RER with KURT; we observed a 3.4% drop (KURT = 0.87, LW = 0.90). RER values under 1.0 confirm submaximal effort. Respiratory frequency increased by 4.3% and 4.6% with KURT vs LW and SW, respectively. While higher respiratory frequency may suggest increased metabolic cost (26), all other variables indicate reduced cost with KURT. This increase in respiratory frequency may relate more to cadence (27) and steering complexity (28), which vary across propulsion systems and require differing neuromotor coordination.

Several limitations should be acknowledged. First, all participants were young, able-bodied individuals. Their biomechanics and cardiopulmonary profiles may not reflect those of regular wheelchair users, especially people with long-term disabilities or joint restrictions. In addition, only males were included, which further limits the generalizability of the findings to female users. One participant was left-handed, which may have introduced minor asymmetries; however, his values were within the range of the other participants, so his data were retained to avoid reducing the sample size. Another limitation is the amount of missing EMG data for some muscles. In several trials, the EMG sensors interfered with the wheelchair frame or wheels, leading to detachment or substantial motion artefacts, so these recordings were excluded from the analysis. As far as the left flexor carpi radialis is concerned, which had valid recordings in fewer than half of the participants across all configurations, the problem was compounded because the sensor was damaged partway through the experiment and could not be used for subsequent measurements. Consequently, results for flexor carpi radialis, especially on the left side, should be interpreted with caution because of the reduced sample size and higher risk of bias in these estimates. Furthermore, participants were allowed to choose their own pace. Although this made the task more realistic and representative of everyday wheelchair use, it resulted in slight variations in circuit completion time. Notably, KURT took longer to complete the circuit, likely due to the higher coordination demands associated with crank propulsion. This suggests users may require a training period to fully master KURT. These timing differences could also have introduced slight variations in resistive power during propulsion.

Despite these limitations, previous studies consistently show that crank-propulsion systems such as hand bikes are more metabolically efficient than push-rim wheelchairs (6, 10, 29). These differences become more pronounced under higher resistance conditions (10). It is therefore possible that using a more demanding task in our experiment might have produced even greater performance differences between KURT and the LW/SW setups.

Large standard deviations in some datasets may be attributed to the use of a single wheelchair setup for all participants. Variations in individual body size and proportions may have led to different joint angle trajectories, possibly exceeding normal physiological ranges and prompting compensatory muscular activity.

From a clinical perspective, the combination of an overall lower cardiopulmonary response with KURT, a more even redistribution of upper-limb muscle activity and operation within a joint range closer to the physiological range of upper-limb motion suggests that handle-based propulsion may reduce cumulative loading on vulnerable shoulder structures and lower long-term overuse risk compared with conventional push-rim propulsion (9). These characteristics make KURT a promising alternative for individuals with limited mobility, pain, or impaired motor function, and particularly for users with existing shoulder complaints or restricted range of motion. Future studies should therefore include members of both sexes and also experienced manual wheelchair users, examine longer adaptation periods and higher resistance levels, and assess long-term use in real-life settings to determine whether the acute biomechanical and metabolic differences observed here translate into reduced pain, improved participation, and sustained joint protection.
